# M1-like, but not M0- or M2-like, macrophages, reduce RSV infection of primary bronchial epithelial cells in a media-dependent fashion

**DOI:** 10.1371/journal.pone.0276013

**Published:** 2022-10-13

**Authors:** Natalie J. Ronaghan, Mandy Soo, Uriel Pena, Marisa Tellis, Wenming Duan, Nooshin Tabatabaei-Zavareh, Philipp Kramer, Juan Hou, Theo J. Moraes

**Affiliations:** 1 STEMCELL Technologies Canada Inc., Vancouver, British Columbia, Canada; 2 Program of Translational Medicine, Hospital for Sick Children Research Institute, Toronto, Ontario, Canada; 3 Department of Laboratory Medicine and Pathobiology, University of Toronto, Toronto, Ontario, Canada; Eötvös Loránd Research Network Biological Research Centre, HUNGARY

## Abstract

Respiratory syncytial virus (RSV) is a common childhood infection that in young infants can progress into severe bronchiolitis and pneumonia. Disease pathogenesis results from both viral mediated and host immune processes of which alveolar macrophages play an important part. Here, we investigated the role of different types of alveolar macrophages on RSV infection using an *in vitro* co-culture model involving primary tissue-derived human bronchial epithelial cells (HBECs) and human blood monocyte-derived M0-like, M1-like, or M2-like macrophages. It was hypothesized that the *in vitro* model would recapitulate previous *in vivo* findings of a protective effect of macrophages against RSV infection. It was found that macrophages maintained their phenotype for the 72-hour co-culture time period and the bronchial epithelial cells were unaffected by the macrophage media. HBEC infection with RSV was decreased by M1-like macrophages but enhanced by M0- or M2-like macrophages. The medium used during the co-culture also impacted the outcome of the infection. This work demonstrates that alveolar macrophage phenotypes may have differential roles during epithelial RSV infection, and demonstrates that an *in vitro* co-culture model could be used to further investigate the roles of macrophages during bronchial viral infection.

## Introduction

Respiratory syncytial virus (RSV) is a filamentous enveloped, negative-sense, single-stranded, RNA virus responsible for common childhood illness. In very small infants or immunocompromised adults, RSV infection can progress to severe bronchiolitis and pneumonia, and is the leading cause of hospitalization of infants under 1 year of age in North America [1]. While deaths in North America are relatively low and decreasing with improved detection and treatment [2], in developing countries, over 100,000 infant deaths were attributed to RSV in 2015 [3]. Infection can also predispose patients to wheezing or asthma [4]. There has been a resurgence of RSV after COVID-19 pandemic restrictions were lifted in Australia and in some US states, causing increased burden on the health care system [5]. Currently there is one FDA-approved monoclonal-antibody for RSV, palivizumab (Synagis), which is used as passive immunoprophylaxis; however use is limited to high-risk infants due to costs, and this approach has no efficacy as a therapeutic [6].

*In vitro*, RSV can infect primary tissue-derived nasal and bronchial epithelial cells but does not cause monolayer damage or change in barrier function [7–10]. Thus, the damage caused *in vivo* by RSV is likely a combination of viral-mediated and host immune response pathology [11]. RSV can enter cells using insulin-like growth factor-1 receptor (IGF1R), which is primarily expressed by ciliated epithelia, and utilizes nucleolin [12, 13]. Pattern recognition receptors in the epithelia detect viral components such as double-stranded RNA and signal downstream to release type I and type III interferons.

After infection follows a cascade of paracrine and autocrine signaling leading to the recruitment of immune cells such as monocytes into the infected area [14]. Monocytes then differentiate into macrophages, and depending on the cytokine environment, into specific macrophage subtypes [15, 16]. Upon resolution of inflammation, macrophages also release pro-resolving factors and clean up dead cell debris. Macrophage phenotype can be identified by unique cell surface markers and cytokine secretion [17, 18]. Although an oversimplification, proinflammatory macrophages are designated as M1-like, while pro-resolution macrophages are M2-like. There are two sources of macrophages in the lung, and each may play unique roles during viral infection [19]. First, alveolar macrophages are yolk sac-derived tissue resident macrophages that sit in the alveolar space. These have the capacity for self-renewal, and act as sentinels to maintain immune tolerance [19]. Alveolar macrophages express CD11c and tend to be more M2-like [20, 21]. Second, interstitial macrophages, which reside in the lung parenchyma, are initially formed in the bone marrow as monocytes and recruited to the lung by appropriate inflammatory stimuli where they differentiate into macrophages [22].

To determine the role of macrophages in RSV infection, *in vivo* models have typically been used that deplete alveolar macrophages, or adoptively transfer specific types of macrophages into the lungs again after depletion. However, there are conflicting findings in these reports, with some suggesting macrophages are protective [23–26], while others suggest they contribute to the pathology [27, 28] of infection. To add to the complexity, modifying the phenotype of alveolar macrophage during infection may also provide a protective effect against viral infection [29, 30]. The use of mouse models to represent human airway diseases or as infection models may limit translatability of findings, as murine lung development, structure, recovery after injury, and the recruitment of immune cells are different than in humans [31, 32].

Thus, the aim of this work was to optimize methods for the co-culture of primary tissue-derived human bronchial epithelia (HBECs) with primary blood-derived macrophages of different phenotypes as a non-contact, paracrine model, and to then determine the role of these macrophages in the epithelial response to RSV infection. We hypothesized that the growth factors in PneumaCult™ ALI Medium, used to differentiate and maintain the HBECs, may polarize the macrophages to a more M2-like phenotype, similar to unstimulated tissue-derived alveolar macrophages. It was also hypothesized that M1 and M2 macrophages would play a protective role and cause a reduction in RSV infection in the epithelia.

We found that only M1-like macrophages considerably decrease the initial infection of HBECs to RSV. Conversely, naïve (M0-like) and M2-like macrophages potentiated RSV infection. We also found that while PneumaCult™ ALI Medium does not induce a significant change in the macrophage polarization by itself or in co-culture with HBECs, during RSV infection this medium significantly impacted the ability of M1 macrophages to reduce the infection. Together, these results demonstrate that different macrophage subsets, influenced by their microenvironment, play different roles during RSV infection.

## Methods

### Primary tissue-derived human bronchial epithelial cells

Materials were obtained from STEMCELL Technologies Inc (Vancouver, Canada) unless otherwise specified. Primary tissue-derived HBECs were obtained from Lonza (Basel, Switzerland) or Epithelix (Plan-les-Ouates, Switzerland), or isolated from whole lung (National Drug Research Interchange, Philadelphia, USA). Eight different donors were used in this study (Table 1).

**Table 1:** Information on primary tissue-derived human bronchial epithelial cells.

Vendor	Cat No.	Lot No.	Age	Sex
Epithelix	EP51AB	680.01 F2	71	F
Epithelix	EP51AB	660.01	2	F
Epithelix	EP51AB	AB534.01	62	F
Epithelix	EP51AB	AB372.01	78	M
Lonza	CC-2540S	0000493462	29	M
Lonza	CC-2540S	0000482214	62	M
Lonza	CC-2540S	0000436081	66	M
NDRI	2103-01627-218	RHOV1 01 001A	91	M

Basal cells were expanded in PneumaCult™-Ex Plus Medium (05040) until 80% confluent, then seeded at 3.3x10^4^ cells/well into 0.4 μm pore size, 0.33 cm^2^ 24-well Corning™Transwells™ (3470, Corning, USA) in PneumaCult™-Ex Plus Medium. After the cells reached confluency, they were air-lifted and the medium replaced with PneumaCult™-ALI Medium (ALI) (05001) to induce differentiation. Cells were differentiated at air-liquid interface for at least 4 weeks before being used in experiments. For initial establishment of co-culture methods, three donors were used at passage 3–5. For RSV infection, six donors were used at passage 3.

### Monocyte isolation and differentiation into macrophages

**Fig 1:**
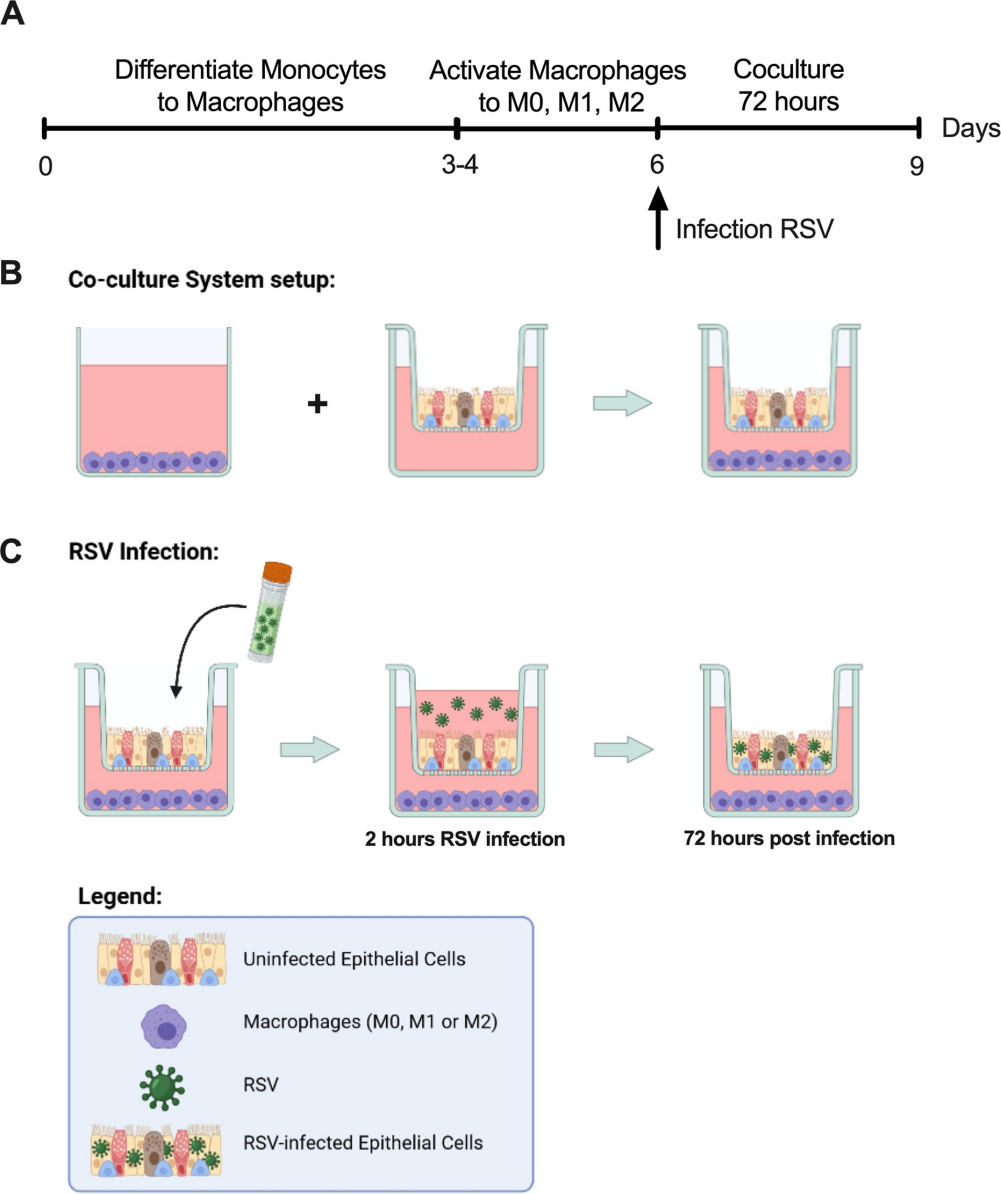
Methods for macrophage differentiation, co-culture, and RSV infection. **(A)** The timeline of monocyte differentiation into macrophages, then into M0, M1-like, or M2-like macrophages. Human blood-derived monocytes were isolated from enriched leukapheresis product and cultured in IMM. Monocytes were differentiated into naive (M0-like) macrophages using IMM with 50 ng/mL M-CSF for 3–4 days, and further differentiated for 2–3 days into M1- or M2-like macrophages through addition of 50 ng/mL IFNγ and 10 ng/mL LPS, or 10 ng/mL IL-4. Cells were then placed in co-culture with human HBECs for 72 hours. In some instances, the epithelial cells were infected with RSV immediately after being placed in co-culture. **(B)** A schematic of the macrophages and HBECs being placed in co-culture. The HBECs were maintained at air-liquid interface and the macrophages were in submerged culture at the bottom of the dish. The macrophages and epithelia were not in direct contact. **(C)** Illustration of co-culture with RSV infection. After cells were placed in co-culture the epithelia were infected by the apical addition of medium containing RSV and incubated for 2 hours; then the inoculum was removed and the epithelia returned to air-liquid interface. The cultures were collected after 72 hours.

Human blood-derived monocytes were isolated from enriched leukapheresis product (200–0131) using EasySep™ Monocyte Isolation Kit (19359), plated in 24-well tissue-culture treated plates (3526, Corning) at 5x10^5^ cells/well, and grown in ImmunoCult™-SF Macrophage Medium (IMM) (10961). Monocytes were differentiated into naive (M0-like) macrophages using IMM with 50 ng/mL macrophage colony-stimulating factor (M-CSF, 78057) for four days. These macrophages were further differentiated for 2–3 days into M1-like macrophages through addition of 50 ng/mL interferon-gamma (IFNγ, 78020) and 10 ng/mL Lipopolysaccharides from Escherichia coli O55:B5 (LPS, L2880-10MG Sigma-Aldrich, Burlington USA). They were also differentiated into M2-like macrophages through addition of 10 ng/mL interleukin-4 (IL-4, 78045). These are M2a-like macrophages but are referred to as M2 macrophages here. The medium to differentiate into M1- or M2-like macrophages also contained 50 ng/mL M-CSF (Fig 1A). All cytokines were human recombinant products. The protocol for differentiation as well as characterization of macrophages using flow cytometry and cytokine release was developed and validated by STEMCELL Technologies Inc [33, 34]. For simplicity, the macrophages will be referred to as M0, M1, or M2 macrophages, “M0 medium” is IMM + M-CSF, “M1 medium” is IMM + M-CSF + LPS + IFNγ, and “M2 medium” is IMM + M-CSF + IL-4, at the concentrations listed above. Each co-culture experiment used a different macrophage donor.

### Set up of co-culture of macrophages with bronchial epithelial cells

Fully differentiated bronchial epithelial cells were placed in the wells containing differentiated (M0, M1, or M2) macrophages. The epithelium was maintained at air-liquid interface throughout the co-culture and the epithelium and macrophages were not in direct contact (Fig 1B). The medium bathing the co-cultures was changed to either ALI medium, M0, M1, or M2 medium. Media, macrophages, and epithelia were assessed 72 hours later.

### Assessment of co-culture after 72 hours

To determine the effect of co-culture on the HBECs, permeability was determined using transepithelial electrical resistance (TER) measurements by voltohmmeter (EVOM) (World Precision Instruments, Sarasota, USA). White light images of macrophages were taken using an Olympus CKX53 microscope with cellSens Software (Olympus Canada, Richmond Hill, Canada) using a 2x objective (PlanN 0.06). Media was removed from the cultures and snap frozen, and release of cytokines determined by ELISAs for TNFα (KHC3011, Thermo Fisher, Hampton, USA), IL-10 (02013), or IL-12p70 (02014). Medium used in ELISAs was diluted 1:5 for the TNFα, but was used undiluted for IL-10 and IL-12p70.

Macrophages were removed from the dish by incubating cells with ACCUTASE™ (07920) (M0 and M1) or 2.5mM EDTA in PBS (E7889 Sigma-Aldrich) (M2) for 20 minutes at room temperature followed by gentle scraping. Any floating macrophages were also included in the analysis. Flow cytometry was performed using a CytoFLEX S Flow Cytometer (Beckman Coulter, Brea, USA) with FCS Express Software (De Novo Software, Pasadena, USA) to determine macrophage polarization. All materials and concentrations are listed in Table 2. Markers used included CD14 (monocyte/macrophage), CD80 and CCR7 (M1-like), and CD206 and CD209 (M2-like). Cells were stained with antibodies for 15 minutes followed by incubation with DRAQ7™ as a live/dead marker. Blocking buffer included anti-CD32 antibody (60012) and 5% Normal Rat Serum (SRT30, Equitech-Bio, Inc, Kerrville, USA). Fluorescence-minus-one (FMO) controls as well as single-stain controls using OneComp eBeads™ Compensation Beads (ThermoFisher Scientific) were used to determine compensation and gating. 10,000 events were recorded per sample. Data was gated to include live cells, and M1 and M2 populations gated against each other, or compared to M0 macrophages (S1 Fig).

**Table 2:** Materials used for flow cytometry of macrophages.

Antibody or Stain Name and ID	Clone	Isotype	Staining Concentration	Vendor	Catalog Number
FITC anti-human CD206 (MMR) (AB_571905)	15–2	Mouse IgG1, κ	2 μg/mL	BioLegend (San Diego, USA)	321104
FITC anti-human CD80 (AB_314502)	2D10	Mouse IgG1, κ	4 μg/mL	BioLegend	305206
APC anti-human CD197 (CCR7) (AB_10917387)	G043H7	Mouse IgG2a, κ	2 μg/mL	BioLegend	353214
APC anti-human CD209 (DC-SIGN) (AB_1134045)	9E9A8	Mouse IgG2a, κ	2 μg/mL	BioLegend	330108
Brilliant Violet 421™ anti-human CD209 (DC-SIGN) (AB_2734323)	9E9A8	Mouse IgG2a, κ	1 μg/mL	BioLegend	330117
Brilliant Violet 421™ anti-human CD14 (AB_10959324)	M5E2	Mouse IgG2a, κ	0.7 μg/mL	BioLegend	301830
DRAQ7™ Live/Dead stain	NA	NA	0.3 μM	BioLegend	424001
OneComp eBeads™ Compensation Beads	NA	NA	10 μL	ThermoFisher Scientific	01-1111-42
Anti-human CD32 Antibody (AB_2722545)	IV.3	Mouse IgG2b, κ	1 μg/mL	STEMCELL Technologies	60012
Normal Rat Serum	NA	NA	5%	Equitech-Bio Inc.	SRT30

### Respiratory syncytial virus isolation and propagation

Hep2 cells (CCL-23, ATCC, Manassas, USA) were maintained at 37°C in Essential Minimal Eagle’s Medium (EMEM, 320-005-CL, Wisent, St-Bruno, Canada) supplemented with 10% FBS (12484–028, Gibco, ThermoFisher). Cells were expanded in T75 flasks (353133, Corning) and infected with a recombinant strain of RSV-A2 expressing green fluorescent protein (rgRSV224, subsequently referred to as RSV) [8, 35] obtained from Mark Peeples’s laboratory (The Research Institute at Nationwide Children’s Hospital, Columbus, USA). RSV was collected from infected Hep2 cells using freeze-thaw cycles, combined with virus released into the medium, and stored in liquid nitrogen. The concentration of virus was calculated as fluorescence focus units per mL (ffu/mL) through serial dilution and infection of Hep2 cells in a 96-well assay. Multiplicity of infection (MOI) was calculated by determining the number of bronchial epithelial cells per Transwell® insert, averaged at 1 million/Transwell® insert.

HBECs were differentiated as described above for more than 4 weeks, washed with PBS, and infected by addition of 0.15 MOI RSV to the apical chamber for 2 hours. The inoculum was then removed. Infection was visible after 48 hours and robust infection in the majority of epithelia was observed after 72 hours at this MOI (S1 Fig).

### RSV infection of co-cultures of HBECs and macrophages

To determine the effect of macrophages on infection with RSV, HBECs were placed in co-culture as described above with M0, M1, or M2 macrophages, and infected with 0.15 MOI RSV (Fig 1). The media in co-culture was changed to ALI, IMM with no cytokines, IMM with individual cytokines (M-CSF, LPS, IFNγ, or IL-4), or M1 or M2 medium. Controls included an uninfected culture in ALI, and infection without macrophages.

Cultures were collected as described above, with some modifications. Fluorescence images were taken with an Olympus IX51 microscope using a 2x objective (Plan Apo 0.08 BF) and white light images of macrophages were taken using a 10x objective (CplanFL N 0.3 Ph3). A 1000x1000 pixel (2.93x2.93 mm) region of interest (ROI) in the center of the image was analysed using ImageJ Software for total fluorescence signal.

Media was collected from the basolateral side of cultures at 24 and 72 hours post infection, and the apical side of the epithelia was washed with 100 μL of PBS and collected. The media and apical wash, which might contain RSV, was treated with 1% Triton X-100 to lyse any potential viral particles, then snap frozen. Media and apical wash were assessed for cytokine secretion using Meso Scale Discovery V-PLEX Proinflammatory Panel 1 (human) (K151A9H-2, Meso Scale Diagnostics, Rockville, USA) for TNFα, IL-10, IL-12p70, or IL-6. Meso Scale Discovery cytokine assay (MSD) was performed as per manufacturer’s instructions but with an overnight incubation of the media to enhance binding to the plate. TER was measured as described above. The macrophages were collected and stained for flow cytometry, then fixed with 4% paraformaldehyde (PFA, 28908, ThermoFisher) for 15 minutes to inactivate RSV. CD80 and CD209 antibody staining performed identically in fixed vs unfixed macrophages but CD14, CCR7 and CD206 were incompatible with fixation.

RNA was collected from the epithelia using EasySep™ Total Nucleic Acid Extraction Kit (STEMCELL Technologies), and cDNA was generated by reverse transcription of 750 ng RNA using Applied Biosystem’s High-Capacity cDNA Reverse Transcription Kit (Waltham, USA) as per manufacturer’s instructions. For qPCR, technical triplicates of 6 μL reactions (for use in Applied Biosystems ViiA 7 Real-Time PCR 384-well system or QuantStudio™7 384-well System [4368814, ThermoFisher]) were prepared with 1 μL of 2X or 300X diluted cDNA. All primers were PrimeTime™ Standard probes from Integrated DNA Technologies (Coralville, USA) (Table 3) and RSV primers were custom-designed using a previously validated sequence for NS1 [36] (Table 4). TaqMan™ Fast Advanced Master Mix was used (4444556, ThermoFisher) as per manufacturer’s instructions. The qPCR program was the following: 1. 95°C for 3 minutes, 2. 40 cycles of 95°C for 15 seconds then 60°C for 1 minute, 3. 4°C hold.

**Table 3:** Predesigned primer sets for PrimeTime™ standard qPCR assay.

Gene Symbol	Gene Name	Assay ID (IDT)
TBP	TATA Box Binding Protein	Hs.PT.58.39858774
GAPDH	Glyceraldehyde 3-phosphate dehydrogenase	HS.PT.39a.22214836

**Table 4:** RSV primer sequence and probe sequence for PrimeTime™ standard qPCR assay.

	Sequence
RSV-NS1 F	5’-AGAGATGGGCAGCAATTCAT-3’
RSV-NS1 R	5’-ACTGGCATTGTTGTGAAATTGG-3’
Probe	5’-/56-FAM/TGCATTGGC/ZEN/TAAGGCAGTGATACATACA/3IABkFQ/-3’

Relative gene expression (ΔCT) was calculated based on the average cycle (Ct) value of the technical triplicates, normalized to housekeeping gene GAPDH or TBP as control. Subsequently, the average ΔCT (n≥3) are reported as fold change (2^(−ΔΔCT)), with a fold change of 1 being assigned to the condition without RSV infection, or medium condition with IMM. TBP was used as a second housekeeping gene but no differences were found between TBP and GAPDH.

### Statistical analysis

Data is assumed to be normally distributed and if displayed in bar graphs represent means ± SE. For statistical analysis, ANOVA was performed using GraphPad Prism version 9.2.0 (GraphPad Software Inc, San Diego, USA). Details are provided in each accompanying figure legend. An associated probability (P) value of <0.05 was considered statistically significant.

### Ethics statement

All human primary tissues and cells used in these studies were obtained from commercial sources (Epithelix, Lonza, and NDRI). Each lot of cells came from a different donor and with a certificate of analysis, verifying that they were derived from donated human tissue after obtaining permission for their use in research applications by informed consent or legal authorization. All donors are anonymized.

## Results

### Co-culture with HBECs and ALI medium caused morphological changes of macrophages with no change in barrier function of HBECs

**Fig 2:**
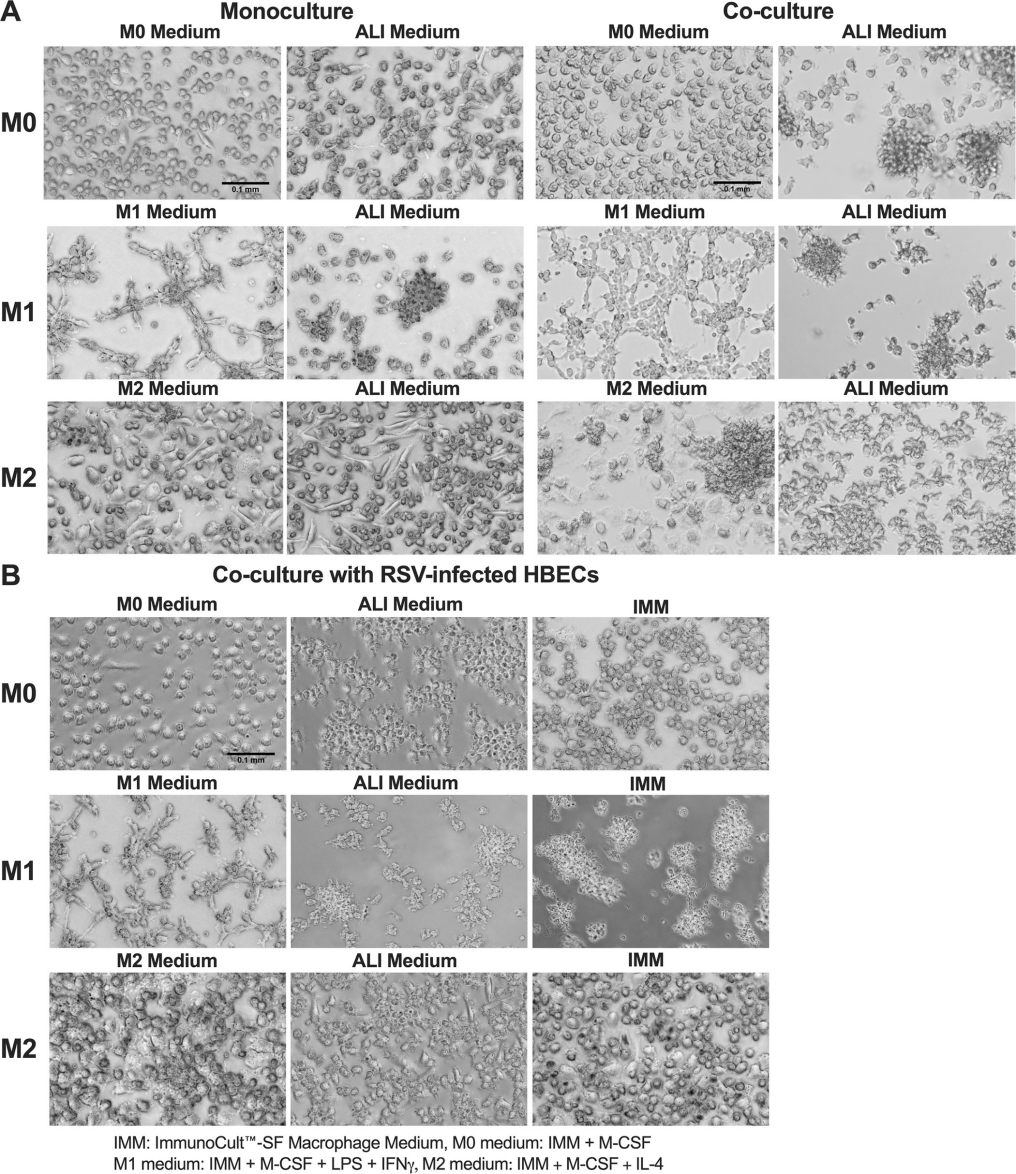
Morphology of M0, M1, or M2 macrophages after culture. **(A)** M0, M1, or M2 macrophages were placed in either M0, M1, or M2 medium (respectively) or ALI medium as either monoculture or co-culture with HBECs for 72 hours and white light images taken to assess morphology. **(B)** M0, M1, or M2 macrophages were placed in either M0, M1, or M2 medium (respectively), ALI medium, or IMM as co-culture with RSV-infected HBECs. Images were taken after 72 hours of co-culture with infected cells and are a representative image of n = 5. Scale bar represents 0.1 mm.

**Fig 3:**
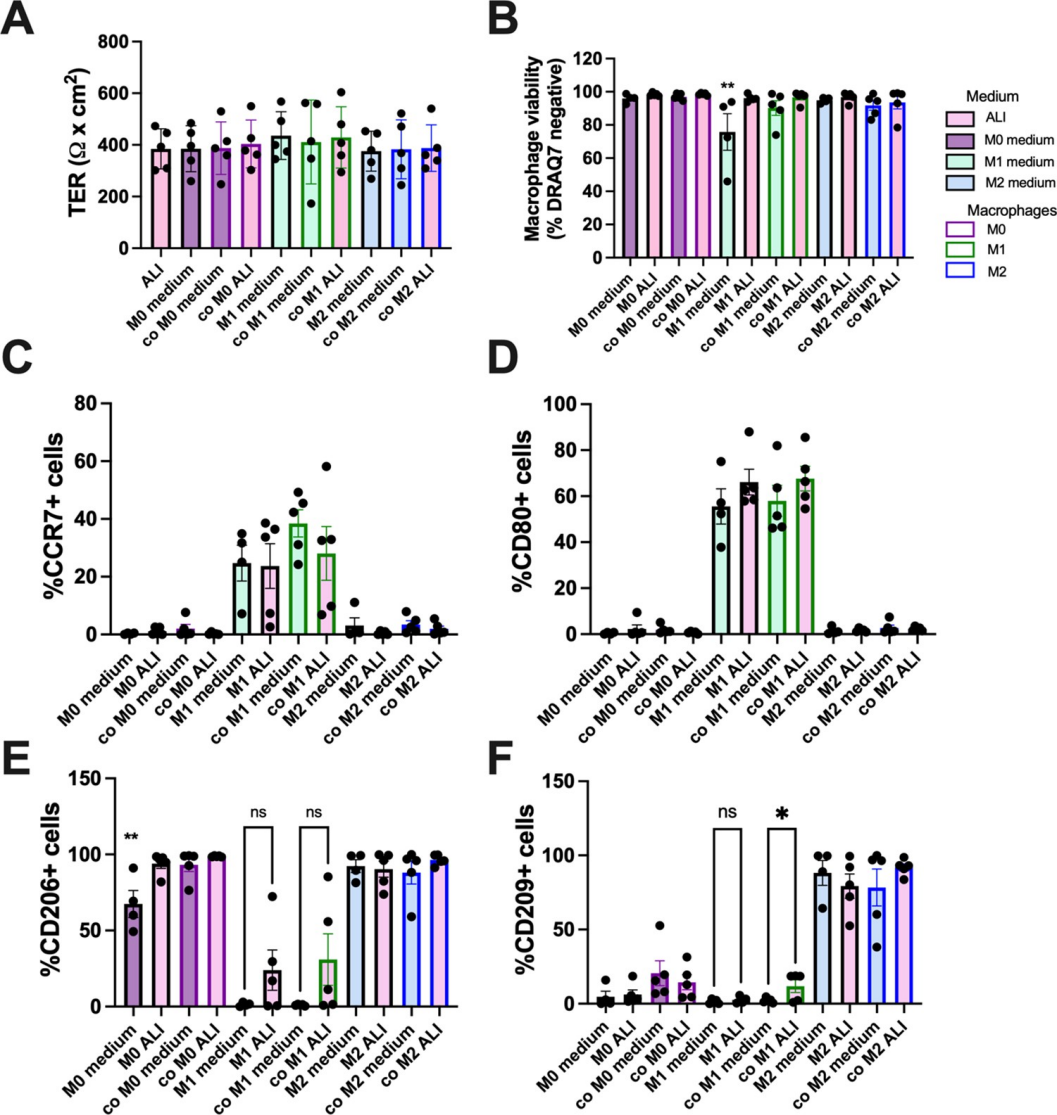
Assessment of phenotypic changes after co-culture of macrophages with HBECs. **(A)** TER of HBECs was measured after co-culture with macrophages or as monoculture in different medium types (M0, M1, or M2 medium, or ALI medium) for 72 hours. In this figure and the subsequent figures, each medium type is represented by a different colored bar, and if they are in co-culture the bar outline color corresponds to the macrophage type. No significant differences in TER were found as determined by ANOVA, n = 5. **(B)** Macrophage viability after monoculture in respective media (M0 medium, M1 medium, M2 medium for the corresponding macrophage type) or in ALI medium, or in co-culture (co) with HBECs in either medium. Viability was determined by flow cytometry by exclusion of DRAQ7™ dye. ** = p<0.01 ANOVA with Tukey’s post hoc test compared to M0 in M0 medium, n = 4–5. **(C-F)**. Flow cytometric analysis of cell surface marker expression for M1 markers (CD80, CCR7) or M2 markers (CD206, CD209) in macrophages after monoculture or co-culture in M0, M1, or M2 medium, or in ALI medium. P<0.05 as determined by ANOVA with Tukey’s post hoc tests (n = 4–5) where *p = <0.05, ** = p<0.01 as determined by ANOVA followed by Tukey’s post hoc test comparing M0 cultures, or where indicated, n = 4–5.

After 72 hours in culture, the macrophages were examined qualitatively for morphological changes [37, 38], rounding or lifting from the plate, or evidence of apoptosis such as blebbing or cell debris (Fig 2). Cells in monoculture in the different media were compared to co-culture with HBECs. In monoculture in M0 or M2 media respectively, M0 and M2 macrophages were rounded and resembled fried eggs. M1 macrophages in M1 medium were elongated and tended to cluster together. In monoculture in ALI medium, some M0 and M2 macrophages detached from the plate after 72 hours or were clustered slightly more. M1 macrophages in ALI medium were less elongated, but remained in clusters. In co-culture with HBECs there were no obvious morphological differences in M0 or M1 macrophages when maintained in M0 or M1 medium, respectively. Co-culture in ALI medium caused exaggerated clumping and lifting of M0 and M1 macrophages. Co-culture of M2 macrophages in M2 medium also caused some clumping, but in ALI medium the macrophages were more similar in appearance to cells in monoculture. There were no changes in transepithelial electrical resistance (TER) (Fig 3A), of the HBECs when incubated for 72 hours in any of the macrophage media, and there were also no changes in TER when in co-culture with macrophages. Thus, the media and co-culture had no significant effect on the health of the epithelia.

### Co-culture with HBECs did not affect macrophage viability or phenotype

Since some of the macrophages lifted from the plate during co-culture, both the floating cells and adherent cells were collected and viability determined by flow cytometry using DRAQ7 viability stain (Fig 3B). M1 macrophages in M1 medium had significantly lower viability (mean viability 75.78% ± 11.01%, n = 4–5) compared to M0 or M2 macrophages (between 91–99% viable). The drop in viability in M1 macrophages was attenuated by culture in ALI or in co-culture in both M1 medium and ALI medium.

Macrophages were collected and examined for phenotype changes by cell surface marker expression (Fig 3C–3F). M1 macrophages expressed CD80 and CCR7, and there were no significant changes in these markers in ALI medium or in co-culture. CCR7 showed some variability between the experiments. M0 and M2 macrophages did not express CD80 or CCR7, and this was not affected by the media or co-culture. M0 and M2 macrophages expressed CD206 and this was unchanged in M2 macrophages in ALI or in co-culture. M0 macrophages expressed significantly more CD206 when in ALI medium or in co-culture. An increase in CD206 was observed by M1 macrophages in ALI medium, although this was not statistically significant. CD209 was expressed by M2 macrophages and this was unchanged by the media or co-culture. There was no difference in CD209 expression by M0 macrophages in co-culture or in ALI medium. CD209 was significantly increased in M1 macrophages in co-culture in ALI medium. There were no significant differences in ALI medium alone, indicating the increase in expression was due to the presence of the epithelium. There was no change in CD209 in co-culture if they were maintained in M1 medium. Thus, each macrophage maintained its cell surface marker expression but ALI medium increased M2 marker expression slightly in M0 and M1 macrophages.

**Fig 4:**
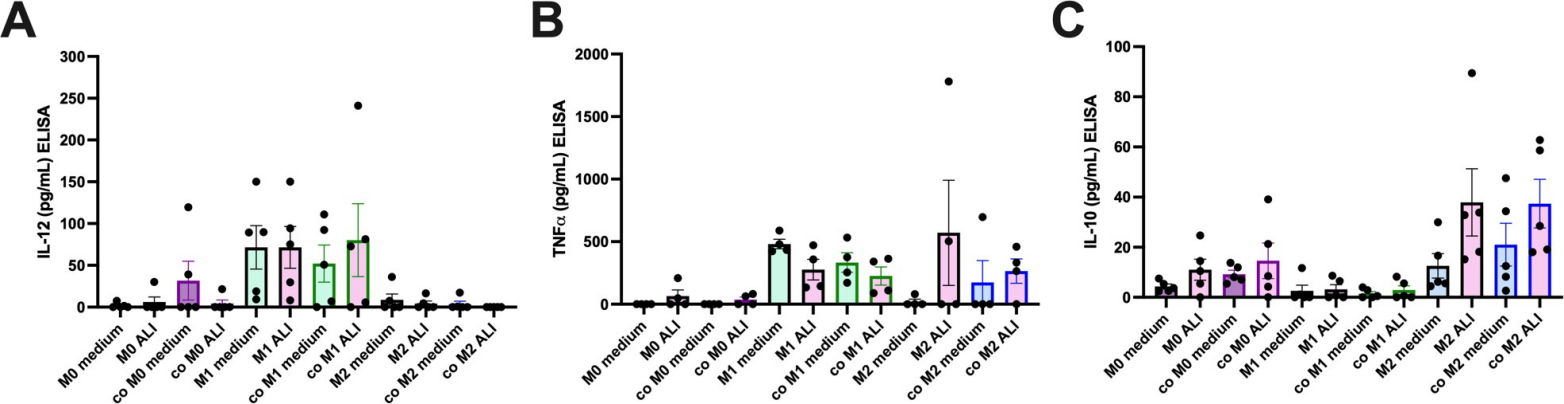
Assessment of phenotypic changes after co-culture of macrophages with HBECs by ELISA. Media was collected after 72 hours of co-culture of macrophages and HBECs and ELISAs for IL-12p70, IL-10, or TNFα were performed. For statistical analysis, ANOVA was performed within each group and compared to monoculture in the medium appropriate for each macrophage (n = 4–5) but no significant differences were found.

Macrophage phenotype in co-culture was also determined by cytokine production by ELISA for IL-12p70 and TNFα (typical M1 cytokines) [18, 39] or IL-10 (a typical M2 cytokine) [40] (Fig 4). The HBECs in M0, M1, or M2 media did not produce detectable amounts of the cytokines examined. M1 macrophages secreted more IL-12p70 compared to M0 and M2 macrophages in their respective media (Fig 4A). M1 macrophages also produced IL-12p70 at similar levels in ALI medium or in co-culture. Neither M0 or M2 macrophages produced significant IL-12p70 and this was unchanged by co-culture or culture in ALI medium. M1 macrophages also secreted TNFα, and there was no significant difference if these macrophages were exposed to ALI medium or were in co-culture for 72 hours (Fig 4B). M2 macrophages did not secrete TNFα but there was a non-significant increase in secretion in some M2 macrophages when exposed to ALI medium or in co-culture. Both M0 and M2 macrophages secreted IL-10 (Fig 4C). There was a trend showing culture in ALI medium resulted in an increase in IL-10 in M2 macrophages, but this was not statistically significant.

### Co-culture of M1, but not M0 or M2 macrophages, caused a decrease in RSV infection as assessed by fluorescent signal

**Fig 5:**
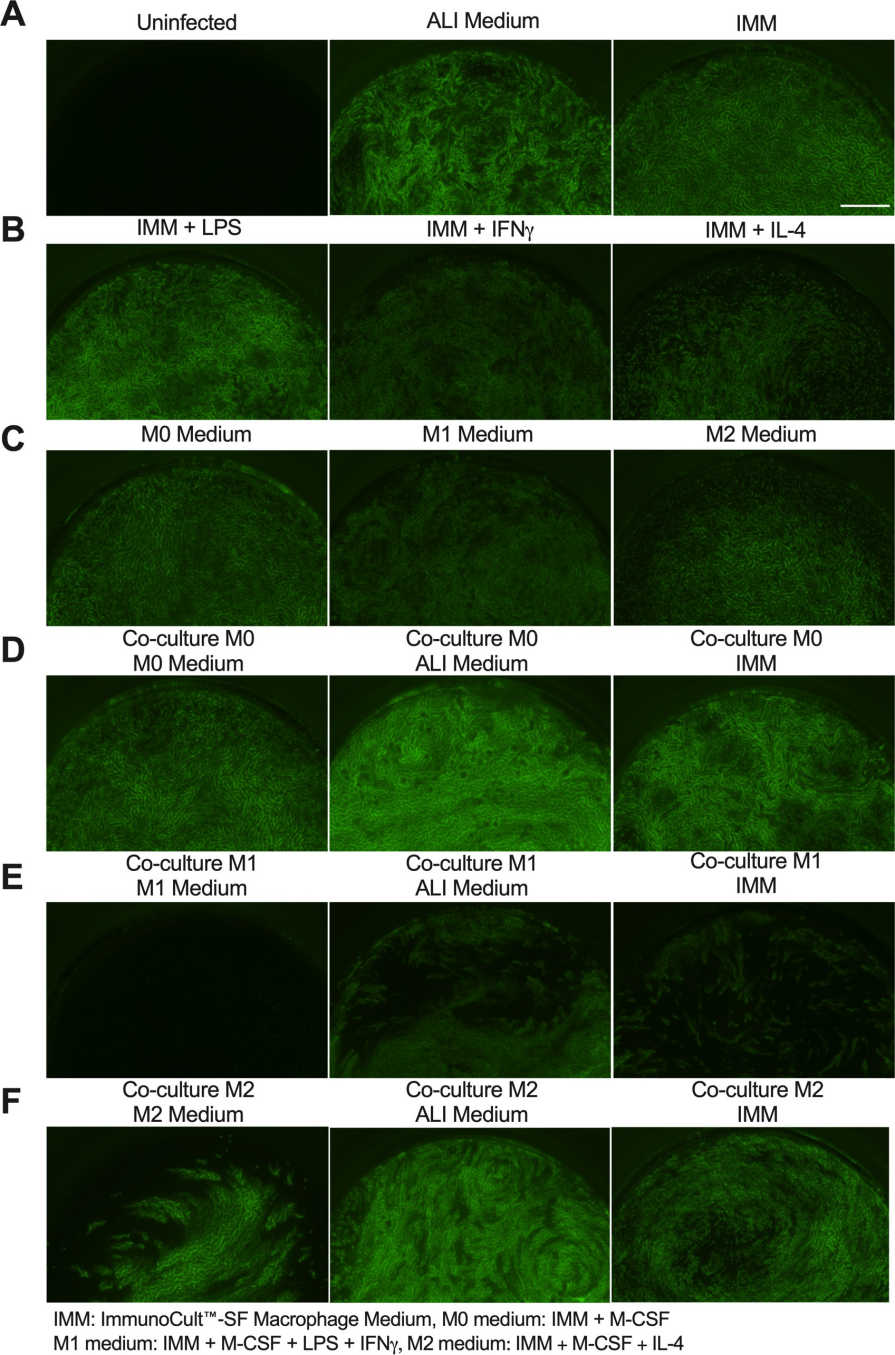
Images of infected HBECs with or without co-culture with macrophages and in different media. HBEC were placed in different types of media as indicated with or without co-culture with M0, M1, or M2 macrophages and infected with 0.15 MOI RSV. 2x images of approximately half of the Transwell area were taken 72 hours post infection. Representative images equal to the average fluorescence as quantified in Fig 5 (n = 3–5). Scale bar represents 1 mm. **(A)** HBECs were uninfected, or infected with RSV when the HBECs were in ALI medium or IMM. **(B)** Media bathing the HBECs was changed to IMM containing either LPS (10 ng/mL), IFNγ (50 ng/mL), or IL-4 (10 ng/mL). **(C)** Media bathing HBECs was changed to M0 medium (IMM with 50 ng/mL M-CSF), M1 medium (IMM with 50 ng/mL M-CSF, 50ng/mL IFNγ and 10 ng/mL LPS), or M2 medium (IMM with 50 ng/mL M-CSF and 10 ng/mL IL-4). **(D, E, F)** HBECs were placed in co-culture with M0, M1, or M2 macrophages and the media changed to M0, M1, or M2 medium, respectively, ALI medium, or IMM.

**Fig 6:**
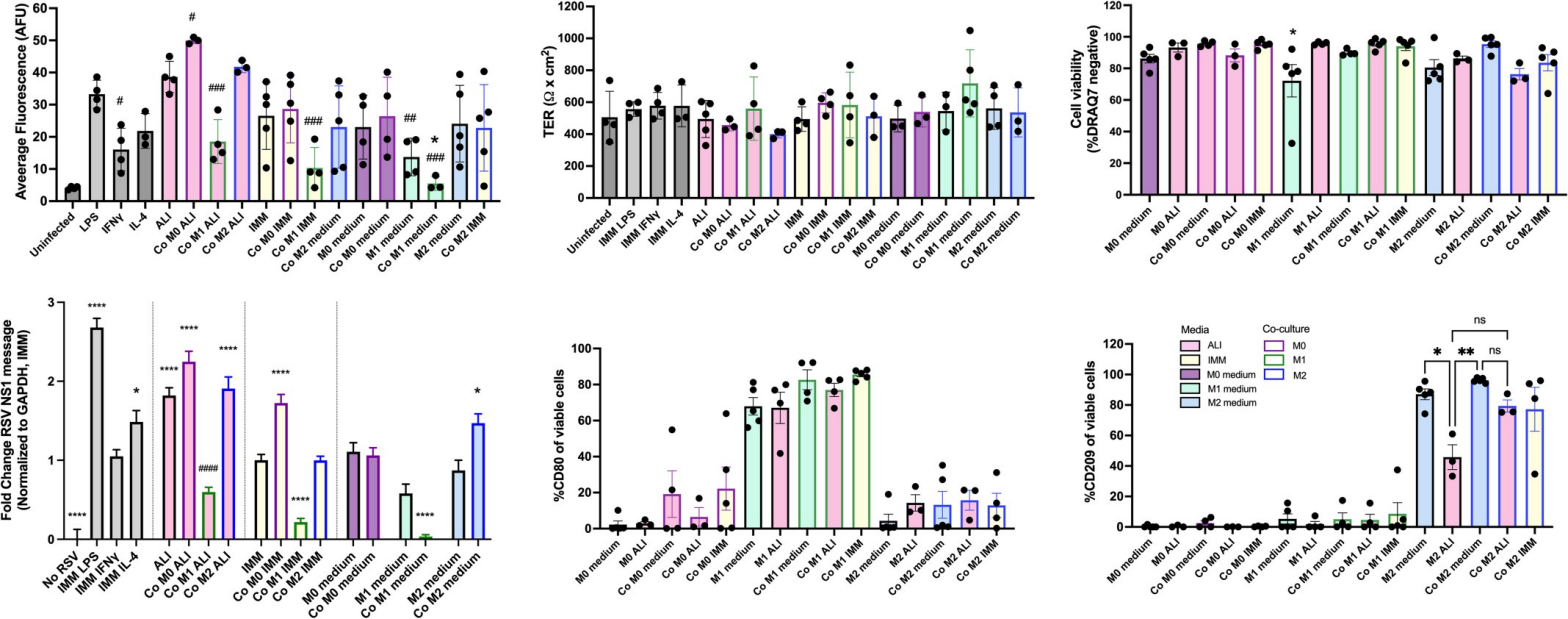
Co-culture with RSV infection. HBECs were placed in different media with or without co-culture with M0, M1, or M2 macrophages and infected with 0.15 MOI RSV. Cells were assessed 72 hours later for **(A)** Average fluorescence of images taken of epithelia, **(B)** Transepithelial electrical resistance (TER) of epithelia, **(C)**, Fold change in RSV NS1 by qPCR (GAPDH as housekeeping gene), **(D)** Macrophage viability as determined by flow cytometry and DRAQ7™ exclusion, or **(E, F)** Macrophage cell surface expression of CD80 or CD206 (M1 or M2 markers, respectively). In B and C, * p<0.05, ** p< 0.01, *** p<0.001, **** p<0.0001 compared to IMM, or # p<0.05, ## p<0.01, ### p<0.001, #### p<0.0001 compared to ALI as assessed by ANOVA with Dunnett’s post hoc test, n = 3–5. In E and F, * p<0.05, ** p< 0.01 as assessed by ANOVA with Tukey’s post hoc test between each macrophage group, n = 3–5.

HBECs were infected with RSV, placed in co-culture at the time of infection, and assessed 72 hours later. Representative images of the fluorescence are shown in Fig 5 and the quantification of this fluorescence is in Fig 6A. There were no statistically significant differences in the infection levels observed based on gender or age of the HBEC donors (n = 6). Co-culture with M1 macrophages in M1 medium caused a significant decrease in fluorescent signal compared to infection in IMM without macrophages. There was also a significant decrease in fluorescent signal in IFNγ alone, or in co-cultures with M1 macrophages in ALI medium or IMM, compared to infection in ALI medium alone. There was no statistically significant effect of M0 or M2 macrophages on fluorescence level.

### RSV infection of co-cultures did not affect TER of HBECs, nor viability and morphology of macrophages

The TER of the epithelia was not affected by the infection or the co-culture with infection (Fig 6B). The viability of the macrophages did not change with co-culture or infection of the epithelia, although a drop in viability of M1 macrophages was observed as in previous experiments. No significant changes in viability were observed in M0 or M2 macrophages (Fig 6D). M0 and M2 macrophages maintained their morphology, while M1 macrophages became more clumped and less elongated (Fig 6B). Similar levels of lifted macrophages were observed with or without RSV infection (Fig 2A).

### As determined by qPCR for RSV NS1, co-culture of M1 macrophages caused a decrease in RSV infection, while M0 and M2 macrophages increased infection, and the effect was dependent on medium type

In addition to the quantification of fluorescence, qPCR was used to determine the level of NS1 message in the infected HBECs as a measurement of viral load (Fig 6C). The fluorescence data correlated with the qPCR data (S3A Fig) and the same trends were observed with a second housekeeping gene, TBP (S3B Fig). The qPCR data was less variable than the fluorescent data.

Based on qPCR measuring RSV NS1, compared to infection in IMM, co-culture with M1 macrophages in IMM or M1 medium significantly reduced RSV infection (Fig 6C). Compared to IMM alone, co-culture of M0 in IMM, or co-culture of M2 in M2 medium, increased RSV infection. As M-CSF alone does not cause a change in infection, the increase due to M0 macrophages in IMM was secondary to the macrophages, and M-CSF may have antagonist effects of the M0 macrophages. There was no change in infection with M2 macrophages in IMM, but IL-4 alone increased infection. Thus, the effect of M2 macrophages in M2 medium may be secondary to the presence of IL-4 in the medium. Interestingly, LPS increased infection in IMM without macrophages, but M1 medium itself did not. There may be opposing effects of M-CSF, LPS, and IFNγ in the M1 medium that can impact infection. ALI medium reduced the ability of M1 macrophages to reduce infection, and this was partially due to the increase in baseline infection levels when the cells are infected in ALI medium, compared to IMM. ALI medium was supplemented with hydrocortisone which may affect polarization of the macrophages [41–43]. However, pilot experiments showed no difference in macrophage phenotype in ALI medium with or without hydrocortisone.

### Co-culture with RSV-infected HBECs did not affect macrophage phenotype as determined by cell-surface marker expression in flow cytometry

The polarization of macrophages was also determined after co-culture with infected cells. As in previous experiments, M1, but not M0 or M2, macrophages expressed CD80 when cultured in M1 medium, and the expression did not change by exposure to ALI medium for 72 hours. Co-culture with RSV infected cells did not change the level of CD80 in any type of macrophage (Fig 6E). CD209 was expressed by M2, but not M0 or M2 macrophages. There were no significant differences in CD209 expression when M2 macrophages were in co-culture with infected HBECs, compared to uninfected cells in the same medium type (Fig 6F).

### Co-culture with RSV-infected HBECs did not affect secretion of IL10, IL-12p70, IL-6, or TNFα as measured by MSD

**Fig 7:**
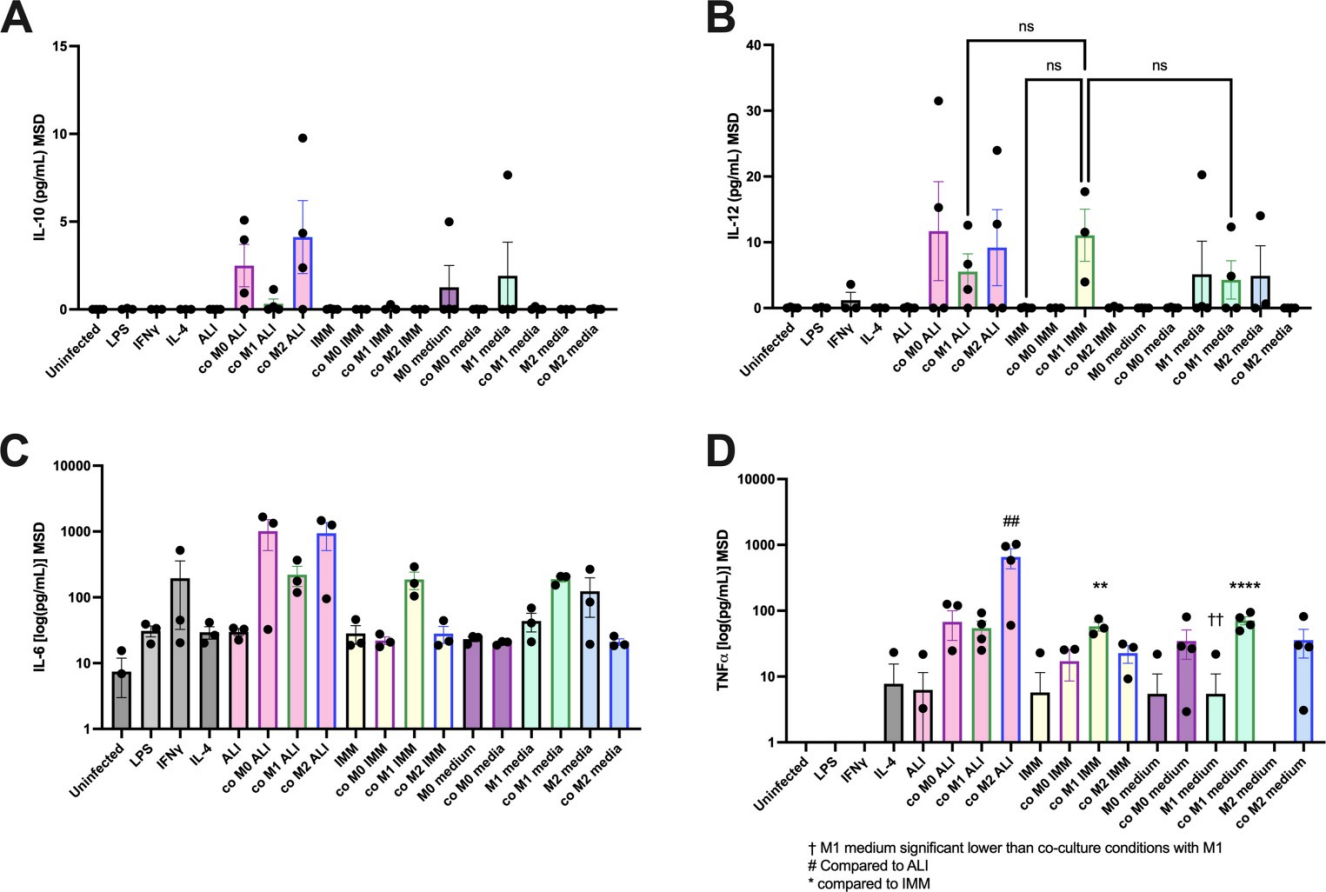
Meso Scale Discovery cytokine assay on media of macrophages and HBECs infected with RSV in co-culture. HBEC were placed in different media with or without co-culture with M0, M1, or M2 macrophages and infected with 0.15 MOI RSV. Media was collected from the basolateral side of the epithelia at 24 and 72 hours post infection and collected. Data for 24 hours is shown, as results at 72 hours were the same. Basolateral media or apical wash were assessed for the release of IL-10, IL-6, IL-12p70, or TNFα by Meso Scale Discovery (MSD) cytokine assay. * p<0.05, ** p< 0.01, *** p<0.001, **** p<0.0001 compared to IMM or where indicated, or # p<0.05, ## p<0.01, ### p<0.001, #### p<0.0001 compared to ALI as assessed by ANOVA with Dunnett’s post hoc test, †† p<0.01 as assessed by ANOVA with Tukey’s post hoc test comparing samples with M1 medium or M1 macrophages, n = 3–4.

The effect of co-culture with infected epithelia on macrophages was also determined by a multiplex cytokine assay from Meso Scale Discovery (MSD), for the release of IL-10, IL-12p70, IL-6, and TNFα at 24 hours or 72 hours post infection into the basolateral media, as well as the release from the apical side. Assessment of the basolateral media for the 24-hour time point only are shown in Fig 7, as results were similar at 72 hours, and no differences in the apical wash were found. After co-culture of macrophages with infected HBECs, very little IL-10 was released, and the highest level was detected in M0 and M2 macrophages in co-culture in ALI medium (Fig 7A). IL-12p70 was increased by infected co-culture with M1 macrophages, or in co-culture of M0 and M2 macrophages in ALI (Fig 7B). There were no statistically significant differences in IL-12p70 secretion from M1 macrophage co-cultures comparing ALI, IMM, or M1 media. Similar to IL-10 production, co-culture of M0 and M2 in ALI medium also produced more IL-6 (Fig 7C). No statistically significant differences were observed for IL-6 release in infected co-cultures.

Compared to infection in IMM alone, there was a significant increase in TNFα observed when the epithelia were co-cultured with M1 macrophages in IMM or in M1 medium (Fig 7D). The increase in TNFα in M1 medium was due to the macrophages, as there was a significant difference between epithelia in M1 medium in monoculture compared to co-culture of M1 macrophages in M1 medium. Co-culture of M2 macrophages in M2 medium also caused an increase in TNFα compared to infection in M2 medium as a monoculture. There was also a significant increase in TNFα release by M2 macrophages in co-culture in ALI medium.

As ELISA and MSD assays have different binding kinetics as well as different standards, M0, M1, and M2 macrophages in M0, M1, or M2 medium, respectively, or incubated in ALI medium for 72 hours were also assessed by MSD as a complementary approach (S4 Fig). These can be compared to the ELISA results in Fig 3. IL-6 was not assessed by ELISA, but MSD demonstrated that M1 macrophages in M1 medium secreted more IL-6 compared to M0 or M2 macrophages in M0 or M2 medium, respectively. In ALI medium, M0 and M2 macrophages secreted more IL-6, although the changes were not statistically significant.

## Discussion

This work describes a well-characterized co-culture model system of primary tissue-derived human bronchial epithelial cells with three types of blood-derived macrophages (naïve M0, proinflammatory cytokine differentiated M1-like, and IL-4 differentiated M2-like macrophages). The co-cultures were then used to determine if these macrophages could affect RSV infection.

Previous groups have developed co-culture models using different epithelia and immune cells, including macrophages. The majority of co-culture methods currently in the literature use immortalized cell lines to represent either the bronchial epithelia (Calu3, A549, BEAS2B) or the macrophages (THP-1, U937) [44–47]. While airway cell lines recapitulate some aspects of the airway, they do not differentiate into mature pseudostratified epithelia with representative cell types such as basal cells, goblet cells, or contain cilia, may not be able to form an air-liquid interface, nor do they respond in the same way to inflammatory stimuli such as a viral infection. Monocyte and macrophage cell lines, while convenient, do not fully represent mature immune cells from the blood, or specific tissue-resident macrophages such as alveolar macrophages. There are some studies that have used both primary tissue-derived bronchial epithelia and macrophages for co-culture, but in these cases the focus was not on the methods and was secondary to the main research goals. For example, Van Riet *et al*. (2020) [48] used a starvation medium in which the co-cultures were incubated in the HBEC medium without supplements. They did not determine if this medium affected the macrophage function. Gindele *et al*. (2017) [49] also used both primary bronchial epithelia and macrophages, but used the medium best suited for macrophages for the co-culture. They noted that this medium did not affect the epithelia but did not include the data in the publication. Similar to the use of animal models, the use of different media may cause conflicting results in co-culture studies, and thus warrants further investigation.

First, we examined whether HBECs could be incubated in medium used for macrophages, which contains M-CSF, LPS, IFNγ, and IL-4 in different combinations. We found that the HBECs were stable with no change in morphology, and the TER did not change after 72 hours in macrophage medium. We also found no difference in TER when HBECs were co-cultured with M0, M1, or M2 macrophages, in either ALI medium or macrophage media. These results are contrary to some studies in epithelial cell lines and airway biopsy samples where cytokines such as IFNγ and IL-4 have been shown to decrease barrier function [50–53]. The media and cytokine combinations used to differentiate macrophages in this work, which always included M-CSF, may not have the same effect on primary tissue-derived HBECs compared to other previously published models. In addition, we use TER only to determine barrier function, which is not representative of changes in barrier function to large molecules [54]. Further characterization of the bronchial epithelia during co-culture, such as permeability to larger molecules (FITC-dextran) to further assess barrier function, ciliary beat frequency, or mucus secretion, was beyond the scope of this work, but may be examined in future studies.

Second, we determined if medium used for the HBECs or co-culture, would affect macrophage viability and morphology. Macrophages were more sensitive to incubation in ALI medium, as morphological changes and lifting from the plate were observed. However, the floating cells were not dead, as determined by viability staining in flow cytometry. There was a slight drop in viability of M1 macrophages in M1 medium, but this was expected due to the presence of pro-inflammatory cytokines that may cause apoptosis in these cells [55]. Culture of M1 macrophages in ALI medium attenuated this viability loss, as the cytokines were removed.

While the morphology changes of the macrophages were visible in ALI medium, there were minimal changes in the phenotype of the macrophages as determined by cell surface marker expression or cytokine release. The few differences were an increase in M2 markers in M0 and M1 macrophages in ALI medium, and a slight but not significant increase in IL-10 secretion in M2 macrophages in ALI medium. Because of the stability of HBECs in macrophage medium with cytokines, and the sensitivity of the macrophages to ALI medium, we did not test additional medium formulations, such as varying combinations or ratios of ALI and macrophage medium.

We chose M0, M1, and M2 macrophages in our co-cultures, but they do not represent the variety of macrophages present in the lungs. They represent extremes of a spectrum, and further studies with other types of macrophages or mixed population may be worthwhile. We used only 4 markers and 2 cytokines to determine the phenotype of the macrophages during co-cultures. In the future additional means to characterize the macrophages such as cytokine arrays, metabolomics, or RNA sequencing may yield interesting associations. We also assumed an M2a phenotype based on CD209 and CD206 expression and IL-10 secretion but did not delve into whether the cells were further differentiated into other M2 subtypes. Overall, the data suggest that co-culture with blood-derived macrophages should prioritize using media supporting the macrophages over the epithelia.

After our investigation of how the media affected the characteristics of the co-cultures, we then determined whether the macrophages could affect RSV infection. We observed robust infection in all HBEC donors after 72 hours by through fluorescent imaging of the GFP-RSV, as well as qPCR for RSV NS1 message. Compared to cell lines, there was the potential for greater differences in infection and increased variability due to heterogeneity of the donors. While we did find some variability in cytokine secretion, the effect of the macrophages on RSV infection as determined by qPCR was consistent. While the qPCR and fluorescent data correlated, the fluorescence data was more variable compared to qPCR. This may be due to limitations of imaging the cells at a macroscopic level to visualize half of the Transwell® insert. In addition, the light may have been scattered from the mucus or pseudostratification of the cells resulting in decreased resolution. It should be noted that we chose to focus on a non-contact co-culture model for these studies. We wanted to understand if the cells could communicate through secreted factors, and then if we could determine which factors may impact infection. Future studies will address other co-culture models, such as apical plating to represent alveolar macrophages, or the basolateral recruitment of monocytes. Future studies could also investigate pre-incubation with macrophages, or adding the macrophages after the infection.

We found proinflammatory M1-like macrophages reduced RSV infection, while pro-resolving M0- or M2-like macrophages increased infection. As evidence suggests alveolar macrophages, through secretion of both type-I [56, 57] and type-II cytokines [28], play roles during RSV infection, we also wanted to determine whether any associations between macrophage phenotype, media for co-culture, RSV infection level, and cytokine secretion could be determined. While IL-12p70 or TNFα, pro-inflammatory cytokines, were secreted from M1 macrophages, we found that both of these cytokines were secreted in similar levels in co-cultures in ALI medium independent of the macrophage type, as well as in co-culture with M2 macrophages. We also measured the release of IL-6 and IL-10 by the co-cultures of infected cells, but no trends were observed. Thus, the question remains as to which cytokines or other signaling molecules may be inducing the effect of M1 macrophages. We did not assess whether the co-culture with macrophages had an effect on the expression of genes in the epithelia related to identifying and eliminating viruses, such as interferon-response genes or the expression of IGF1R. Thus, future assessment of the mechanism, including gene expression using qPCR, RNA sequencing, or more complete cytokine analysis may provide some insight. In addition, although we measured at 24- and 72-hours post infection, assessment at earlier timepoints may provide a more accurate timeline of cytokine release.

In our investigation of co-culture methods prior to infection, it was found the medium used during the co-culture did not significantly affect cytokine secretion by the macrophages. ALI medium did, however, increase the expression of the M2 marker CD206 in both M0 and M1 macrophages slightly. If M1 macrophages are proposed to be pro-inflammatory and reduce RSV infection, then culture with ALI medium, which may reduce M1 phenotype, would be expected to limit the effect M1 macrophages have on the infection. Indeed, co-culture in ALI medium attenuated the effect of M1 macrophages on reducing the infection.

Our work emphasizes that future studies examining co-culture systems should consider how medium can change the function of the cells, and thus the outcome of the experiments. These data may explain why studies *in vivo* depleting macrophages prior to RSV infection have conflicted findings to studies that inhibit macrophage migration or alter macrophage phenotype, as each method might affect different populations of macrophages. Determining which signaling molecules are released by the macrophages to limit infection in epithelia, development of molecules that modify macrophage polarization, or using exogenous application of macrophages could be developed as potential therapeutics for RSV infection.

## Supporting information

S1 Fig Gating strategy for macrophages in flow cytometry. Representative experiment demonstrating the gating strategy after flow cytometry for macrophages. Macrophages were gated in Forward Scatter (Area)-Side Scatter (Area) (FSC-SSC) to exclude debris, then gated as single cells in the FSC-A-FSC-H (Height) plot. Viable cells were gated using DRAQ7™ viability stain. Fluorescence-minus-one and single cells were used to place gates to remove negative staining (not shown). Then, M1 and M2 macrophage control cells were gated against each other so that the M2 gate excluded M1 cells, while the M1 gate excluded M2 cells. M0 macrophages do not express CD80 or CD209. A similar strategy was used for CD206 and CCR7.
(TIF)

S2 Fig Time course RSV infection. HBECs were infected with 0.15 MOI of RSV and infection observed by presence of GFP over 1 week. Scale bar represents 1 mm.
(TIF)

S3 Fig qPCR of infected HBECs to determine potential change in epithelial markers. HBEC were placed in different types of media with or without co-culture with M0, M1, or M2 macrophages and infected with 0.15 MOI RSV. RNA was extracted from the epithelia, reverse transcribed into cDNA, and qPCR used to determine expression of RSV *NS1*. **(A)** Pearson’s correlation indicates a positive correlation between RSV fluorescence vs RSV NS1 message as assessed by qPCR, R2 = 0.7749, p<0.0001. **(B)** The ΔΔCT method was used to determine fold change in expression of RSV *NS1* compared to infection in IMM, with TBP as a housekeeping gene. For RSV, significant differences were found (** p< 0.01, *** p<0.001, **** p<0.0001 compared to IMM, or ## p<0.01, ### p<0.001, #### p<0.0001 compared to the ALI condition as assessed by ANOVA with Dunnett’s post hoc test, n = 3–4).
(TIF)

S4 Fig Meso Scale Discovery cytokine assay on media of macrophages in monoculture. M0, M1, or M2 macrophages were placed in M0, M1, or M2 medium, respectively, or ALI medium for 72 hours. Medium was collected from the cells at 24 hr and 72 hours post media change, and assessed for release of IL-10, IL-6, IL-12p70, or TNFα by Meso Scale Discovery cytokine assay, n = 3. Note the IL-12p70 y-axis is not in log scale. Each n = 3, but some points may be missing on the log scale if the cytokine was undetected.
(TIF)
