# Supplementary figures and images for: M1-like, but not M0- or M2-like, macrophages, reduce RSV infection of primary bronchial epithelial cells in a media-dependent fashion
Source: PLoS One. 2022 Oct 13;17(10):e0276013. doi: 10.1371/journal.pone.0276013 (PMC9560600; doi:10.1371/journal.pone.0276013)

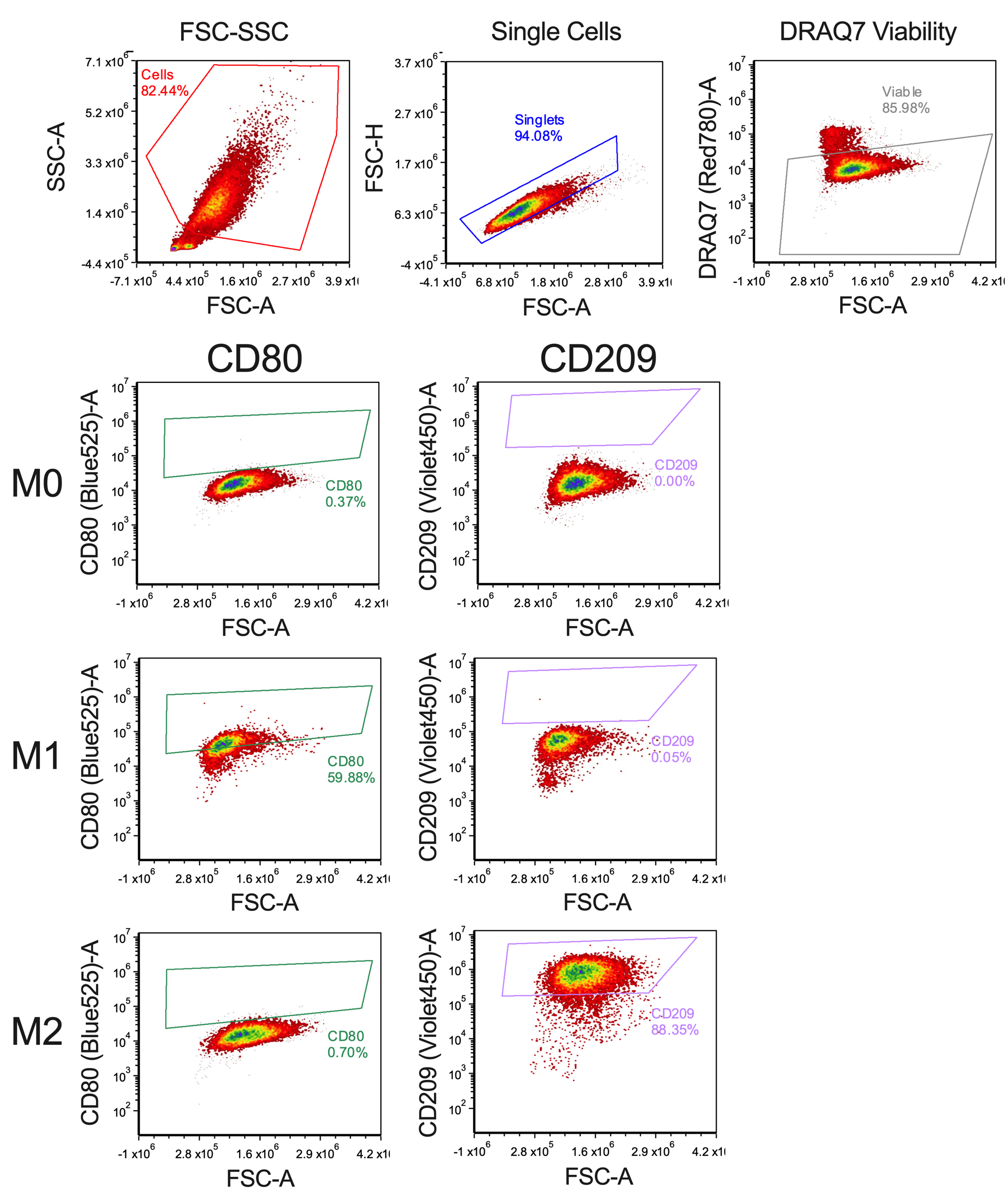

Supplement: S1 Fig — Representative experiment demonstrating the gating strategy after flow cytometry for macrophages. Macrophages were gated in Forward Scatter (Area)-Side Scatter (Area) (FSC-SSC) to exclude debris, then gated as single cells in the FSC-A-FSC-H (Height) plot. Viable cells were gated using DRAQ7™ viability stain. Fluorescence-minus-one and single cells were used to place gates to remove negative staining (not shown). Then, M1 and M2 macrophage control cells were gated against each other so that the M2 gate excluded M1 cells, while the M1 gate excluded M2 cells. M0 macrophages do not express CD80 or CD209. A similar strategy was used for CD206 and CCR7. (TIF) [file pone.0276013.s001.tif]

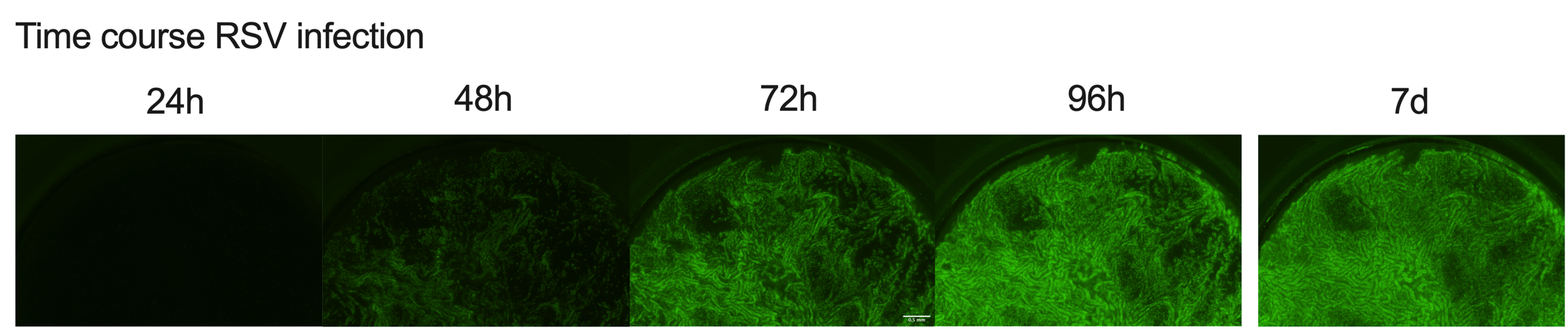

Supplement: S2 Fig — HBECs were infected with 0.15 MOI of RSV and infection observed by presence of GFP over 1 week. Scale bar represents 1 mm. (TIF) [file pone.0276013.s002.tif]

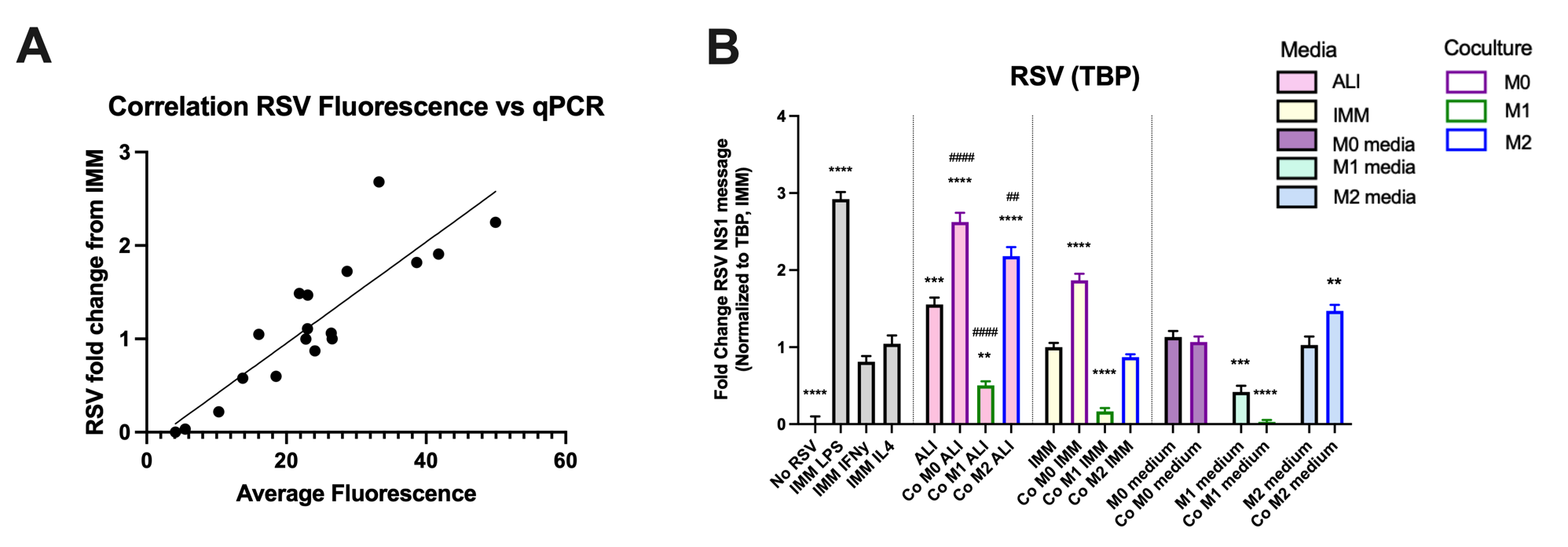

Supplement: S3 Fig — HBEC were placed in different types of media with or without co-culture with M0, M1, or M2 macrophages and infected with 0.15 MOI RSV. RNA was extracted from the epithelia, reverse transcribed into cDNA, and qPCR used to determine expression of RSV NS1. (A) Pearson’s correlation indicates a positive correlation between RSV fluorescence vs RSV NS1 message as assessed by qPCR, R2 = 0.7749, p<0.0001. (B) The ΔΔCT method was used to determine fold change in expression of RSV NS1 compared to infection in IMM, with TBP as a housekeeping gene. For RSV, significant differences were found (** p< 0.01, *** p<0.001, **** p<0.0001 compared to IMM, or ## p<0.01, ### p<0.001, #### p<0.0001 compared to the ALI condition as assessed by ANOVA with Dunnett’s post hoc test, n = 3–4). (TIF) [file pone.0276013.s003.tif]

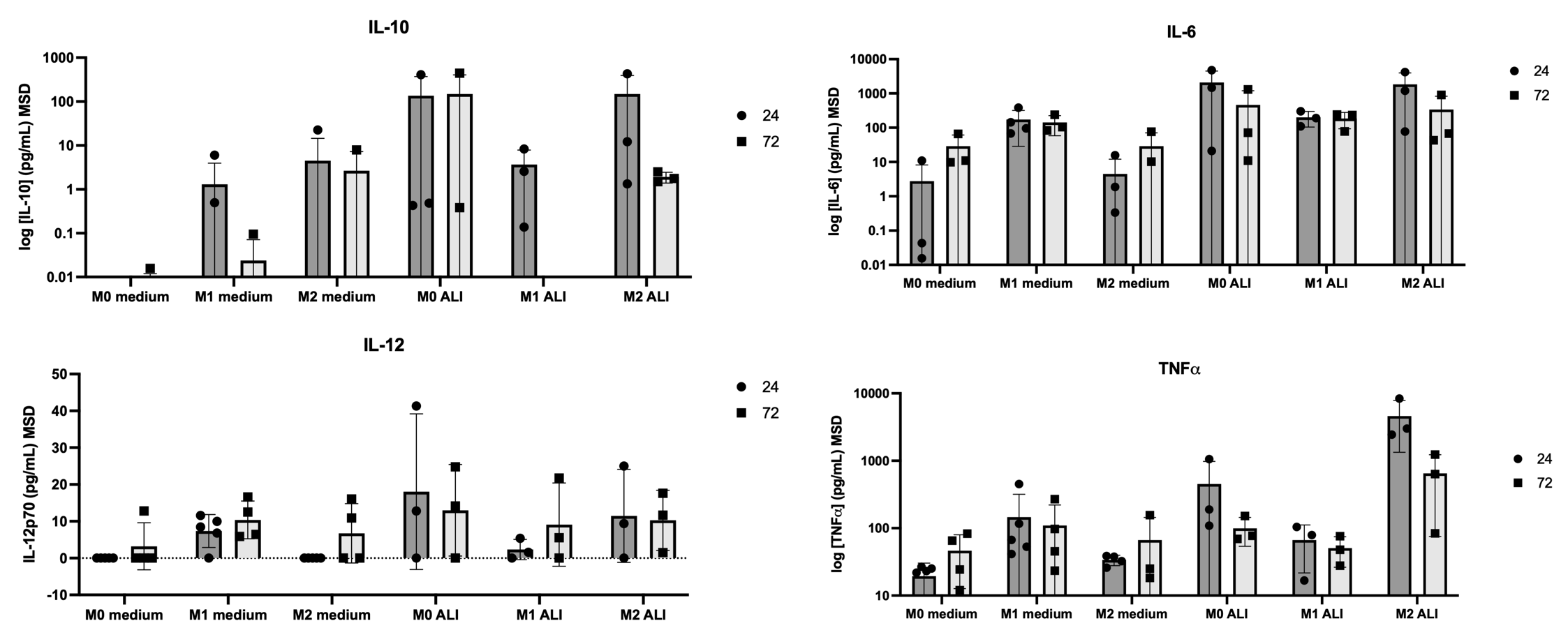

Supplement: S4 Fig — M0, M1, or M2 macrophages were placed in M0, M1, or M2 medium, respectively, or ALI medium for 72 hours. Medium was collected from the cells at 24 hr and 72 hours post media change, and assessed for release of IL-10, IL-6, IL-12p70, or TNFα by Meso Scale Discovery cytokine assay, n = 3. Note the IL-12p70 y-axis is not in log scale. Each n = 3, but some points may be missing on the log scale if the cytokine was undetected. (TIF) [file pone.0276013.s004.tif]
